# Hospital Meetings

**Published:** 1896-07-18

**Authors:** 


### HOSPITAL MEETINGS.

### THE PASSMORE EDWARDS CONVALESCENT HOME.

Charing Cross Hospital is much to be congratulated upon the possession of this beautiful convalescent home, which was opened by the Prince of Wales on Saturday, the 11th inst. It is indebted to the generosity of Mr. Passmore Edwards for nearly the entire gift of the building, and to Mr. Drummond, one of the treasurers, for a site which may be described as ideal. On Saturday a special train conveyed a large party of guests to Oxted to take part in the ceremony. The drive from Oxted station to Limfield Chart, where the home is situated, enabled the visitors to experience the salubrious and invigorating qualities of the air. The route was adorned with charming floral arches and devices to welcome the Royal party, who arrived at twelve o'clock, and after being received by Mr. Passmore Edwards, by the Treasurer of the hospital, and other of the hospital officials, proceeded to the tent erected for the occasion. Here the Prince of Wales, who was accompanied by the Princess of Wales and the Princess Victoria, delivered a brief but very happy speech, in which he commented upon the healthiness of the site chosen. The Bishop of Southwark conducted the opening ceremony, and after the presentation of the architect, the builder, and others connected with the scheme, the Royal party inspected the home, with which they expressed themselves delighted. The building is placed upon a plateau nearly at the top of a slope, which rises above a glorious stretch of valley, and stands 532 ft. above the sea level. The hill which backs the home shelters it from north and east winds, and near at hand is a wood and Limpsfield Common. The grounds are 19 acres in extent. The building is commodiously planned. At either end of the ground floor is a ward, one for men and one for women. Between are offices and sitting-rooms, fine dining hall and kitchen. Above is a children's ward and bed-rooms. On the ground floor, and close to the wards at each end are large covered alcoves, which command the beautiful views, and afford delightful air spaces for the patients in wet weather. The furnishing is neat and suitable, the prevailing colour of walls and draperies a soft terra-cotta; the general effect pleasant and cheerful. The accommodation is for 50 patients, 20 men, 20 women, and 10 children; the entire control and administration is vested in Charing Cross Hospital. A few patients will be received from elsewhere on payment. The home is entirely unendowed. The cost of maintenance will exceed £2,000, and it is hoped that kind friends will be found to take this burden off the hospital. A visit to the Home will convince anyone of the genuine benefit to be obtained by a weakly London patient from residence within it.

